# Interplay between Phytochemicals and the Colonic Microbiota

**DOI:** 10.3390/nu15081989

**Published:** 2023-04-20

**Authors:** Chohee Kwon, Meran Keshawa Ediriweera, Somi Kim Cho

**Affiliations:** 1 Department of Environmental Biotechnology, Graduate School of Industry, Jeju National University, Jeju 63243, Republic of Korea; chkwon222@gmail.com; 2 Department of Biochemistry and Molecular Biology, Faculty of Medicine, University of Colombo, Colombo 008, Sri Lanka; meran@bmb.cmb.ac.lk; 3 Interdisciplinary Graduate Program in Advanced Convergence Technology and Science, Jeju National University, Jeju 63243, Republic of Korea

**Keywords:** phytochemicals, gut microbiota, colonic microbial metabolites, polyphenols, diseases, diversity

## Abstract

Phytochemicals are natural compounds found in food ingredients with a variety of health-promoting properties. Phytochemicals improve host health through their direct systematic absorption into the circulation and modulation of the gut microbiota. The gut microbiota increases the bioactivity of phytochemicals and is a symbiotic partner whose composition and/or diversity is altered by phytochemicals and affects host health. In this review, the interactions of phytochemicals with the gut microbiota and their impact on human diseases are reviewed. We describe the role of intestinal microbial metabolites, including short-chain fatty acids, amino acid derivatives, and vitamins, from a therapeutic perspective. Next, phytochemical metabolites produced by the gut microbiota and the therapeutic effect of some selected metabolites are reviewed. Many phytochemicals are degraded by enzymes unique to the gut microbiota and act as signaling molecules in antioxidant, anti-inflammatory, anticancer, and metabolic pathways. Phytochemicals can ameliorate diseases by altering the composition and/or diversity of the gut microbiota, and they increase the abundance of some gut microbiota that produce beneficial substances. We also discuss the importance of investigating the interactions between phytochemicals and gut microbiota in controlled human studies.

## 1. Introduction

The term holobiont describes the intimate relationship between the host and its microbiota. The gut microbiota has a marked influence on health and disease. Trillions of bacteria (and above 1000 species), archaea, fungi, and viruses reside in the human gastrointestinal tract [1]. Neonates inherit their microbiota from their mother during delivery and breastfeeding [2]. The composition and diversity of the gut microbiota change with age and are influenced by the host’s diet, antibiotics, xenobiotics, infections, genetics, epigenetics, immunity, lifestyle, and environment [3,4,5]. Among these factors, diet has the greatest impact on the gut microbiota [6,7,8,9,10]. The gut microbiota inhabiting the colon metabolizes undigested food material reaching the colon and produces a variety of metabolites which influence the composition of the gut microbiota [11,12]. Microbial metabolites are absorbed by the host and reach almost all organs, influencing the host metabolism and signaling. This dynamic relationship between diet, gut microbiota, and host has resulted in clinical trials of bacterial metabolites and gut bacterial species [13,14,15]. In the present review, we focus on the colonic microbiota since the interaction of the gut microbiota and phytochemicals mostly occurs in the colon.

**Figure 1:**
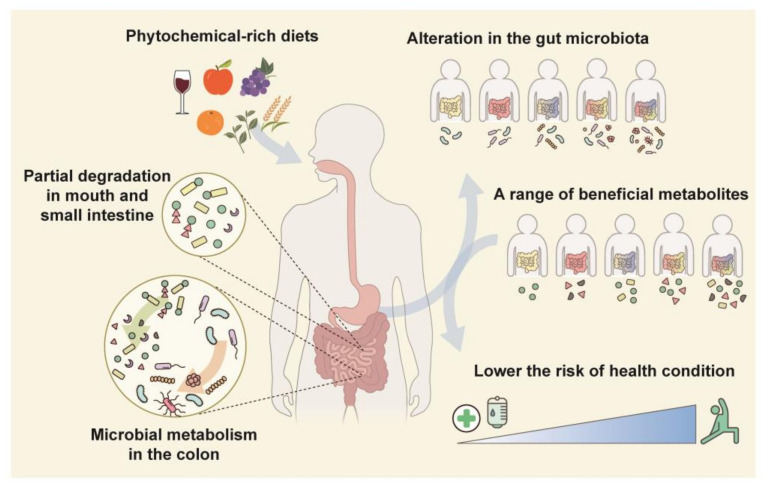
Relationship between the colonic microbiota and phytochemicals. Most phytochemicals in fruits and vegetables are transformed by colonic-microbiota-derived enzymes. Phytochemical metabolites are absorbed into the host circulation and modulate colonic microbiota composition or diversity. An individual’s unique colonic microbiota produces a variety of metabolites which are linked to the risk of several diseases.

Phytochemicals are chemical compounds found in plant-derived foods such as fruits, vegetables, cereals, teas, and mushrooms. Large number of phytochemicals are being researched with the purpose of developing drugs because many are generally safe, abundant in familiar food ingredients, and exert a health-promoting effect [16]. Many phytochemicals have poor bioavailability due to their molecular structure, solubility, stability, and other factors. Metabolism by the colonic microbiota transforms phytochemicals into compounds that are readily absorbable by colonic epithelial cells [17,18]. Such transformed phytochemicals can regulate the redox balance and metabolism and have antioxidant, antimicrobial, anti-inflammatory, and anticancer activities [16,19]. This association with the colonic microbiota causes inter-individual differences in the efficacy of phytochemicals (discussed later in this review). The composition and diversity of the colonic microbiota can be altered by phytochemicals. This alteration is considered to be associated with the amelioration of various diseases (Figure 1) [20,21,22].

Phytochemicals and the colonic microbiota are potential therapeutic candidates for several diseases. Herein, we discuss the bioactivities of phytochemical metabolites produced by the colonic microbiota and highlight the role of the colonic microbiota as a mediator of the therapeutic effects of phytochemicals.

## 2. Role of the Colonic Microbiota in Human Health

### 2.1. Influence on Host Health

The colonic microbiota influences host health by continuously interacting with the host to promote digestion and the absorption of nutrients [23,24]; to produce energy sources [25], hormones [26], neurotransmitters [27], and vitamins [28]; to shape the immune system [29]; and to protect against pathogens and exogenous toxins [30,31]. These activities and their effects on human health are of such significance as to warrant the classification of the colonic microbiota as an organ.

The colonic microbiota influences metabolic homeostasis [32,33]. In C57BL/6J mice, *Akkermansia muciniphila* ameliorated the metabolic endotoxemia by secreting protein P9, which induced glucagon-like peptide-1 secretion by enteroendocrine cells [34]. In a recent a proof-of-concept exploratory study, the safety, tolerance, and metabolic effects of *Akkermansia muciniphila* were investigated. Daily oral supplementation of *Akkermansia muciniphila* for 3 months reduced the total cholesterol level and improved insulin sensitivity [35]. The gut–liver axis plays an important role in metabolic homeostasis. The gut microbiota helps to keep intestinal epithelial cells and tight junctions healthy and protects the liver from inflammatory substances and pathogens [36].

Furthermore, the colonic microbiota is a key component of the gut–brain axis and produces neurotransmitters and other metabolites that affect the brain [37,38]. The brain and the gut bidirectionally interact via central nervous system (CNS), hypothalamic–pituitary–adrenal (HPA) axis, endocrine system, and immune system [39]. Responding to factors such as stress and emotion, the HPA axis secretes several hormones which affect gut motility, digestion, and ultimately the ecosystem of the gut microbiota. Meanwhile, the colonic microbiota produces important neurotransmitters and metabolites such as serotonin and γ-aminobutyric acid (GABA), which modulate emotion and behavior and affect the neuronal signaling, digestive function, and immune system of the host [40]. A recent study showed that dysbiosis in pregnant mice seriously impaired fetal neurodevelopment. Thalamic explants from embryos of antibiotic-treated mothers at embryonic day 14.5 showed impaired axonogenesis; however, the number of axons was increased by treatment with maternal microbial metabolites [41].

The colonic microbiota is also involved in respiratory health [42]. The interaction between the gut and the lung is mostly mediated by the colonic microbiota’s ability to secure the immune system of the host [43]. Its involvement in COVID-19, which is caused by severe acute respiratory syndrome coronavirus 2 (SARS-CoV-2), has been described [44]. SARS-CoV-2 invades upper respiratory cells and leads to asymptomatic, mild, or lethal respiratory infection in addition to gastrointestinal symptoms and gut dysbiosis [44,45,46,47]. Yeoh et al. showed that the abundance of *Faecalibacterium prausnitzii* and *Bifidobacterium bifidum* were inversely correlated with the severity of SARS-CoV-2 symptoms [46]. The reduction of *Faecalibacterium prausnitzii* was inversely correlated with the levels of inflammatory cytokines and post-acute-COVID-19 syndrome [46,48].

As we have seen above, many beneficial effects of the colonic microbiota are due to its immunomodulatory activity [49]. The gastrointestinal (GI) tract is a major site of immune surveillance. The colonic microbiota and its metabolites interact with immune cells in the GI tract; hence they are related to gut barrier function and immune-related GI diseases [50,51]. For example, *Faecalibacterium prausnitzii* is known to exert anti-inflammatory effects in Crohn’s disease and inflammatory bowel disease models by secreting proteins that inhibit the NF-κb pathway [52,53]. In a colon cancer animal model, *Streptococcus thermophilus, Lactobacillus rhamnosus GG*, and *L. gallinarum* exerted anticancer activities by inducing cell cycle arrest and apoptosis or by reducing the expression of inflammatory proteins [54,55]. The gut–heart axis also works through the gut microbiota’s association with the intestinal permeability and immune system [56]. The colonic microbiota reduces the risk of heart failure and coronary artery disease by suppressing inflammation and the total cholesterol level [57].

**Figure 2:**
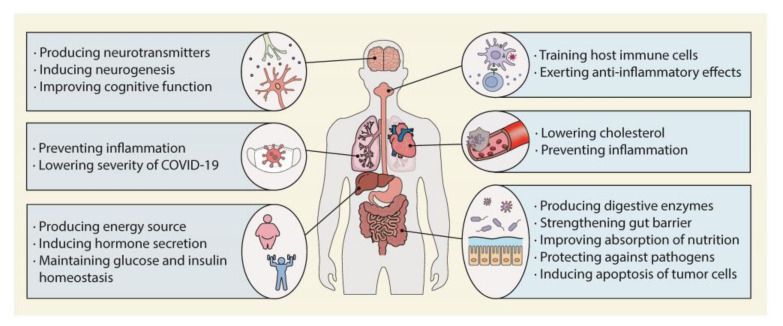
Role of the colonic microbiota in human health. The colonic microbiota can promote human health by affecting almost every organ.

The term dysbiosis refers to an imbalance in the diversity and composition of the colonic microbiota. Dysbiosis is detrimental to the host’s growth and survival [58,59]. Germ-free mice have been shown to live with impaired cardiovascular [58], immunomodulatory [59,60], and neurological functions [61,62,63]. Young germ-free mice showed stunted growth and decreased somatotropic insulin-like growth factor 1 (IGF1) activity. These harmful effects were reversed through the colonization of the GI tract with lactobacilli that activated the somatotropic axis [64]. In a human study, dysbiosis caused by antibiotic treatment during the neonatal period was observed at 24 months old, suggesting a stunting of growth in the first 6 years of life [65]. Furthermore, the colonic bacterial composition was found to be related to the survival of patients with hemodialysis or cervical cancer and even to the survival of healthy seniors [66,67,68]. Notably, fecal microbiota transplantation from patients with obesity, irritable bowel syndrome, colitis, colorectal cancer, or schizophrenia to germ-free mice induced the same diseases or related physiological alterations in the mice [69,70,71,72,73]. Figure 2 illustrates the various roles of the colonic microbiota in the human body. To clarify causality and develop novel therapies, further mechanistic interventional studies controlling for diet, underlying diseases, and other factors affecting the gut microbiome are needed.

### 2.2. Production of Metabolites That Modulate Host Health

Diet is a major determinant in shaping the gut microbiota composition, diversity, and/or metabolic function [74,75,76,77]. The significance of diet is well-established in several studies which observed the change in the gut microbiota through a comparison of the Mediterranean diet (a healthy dietary pattern in the Mediterranean region which is composed of vegetables, fruits, fish, cereals, legumes, and olive oil) and the Western diet (which is rich in fat, proteins, and sugars) [78]. In a recent human study, 14 days of the Mediterranean diet did not significantly change the gut microbial composition but did change the microbial metabolic pathways when compared to the Western diet [79]. A long-term Mediterranean dietary lifestyle was highly associated with an increased level of SCFA-producing bacteria and a decreased level of L-*Ruminococcus* (*Ruminococcus* genus of *Lachnospiraceae* family), which is related to a high intake of fat and sugar [80].

The gut microbiota generates beneficial metabolites from the host diet. Using metagenomic shotgun sequencing, Visconti et al. analyzed blood samples from 859 individuals. Of 673 annotated metabolites in samples from more than 50 individuals, 309 (46%) were associated with the gut microbiota [81]. Several studies have evaluated the roles of gut microbial metabolites in human diseases [82,83,84,85]. Inosine, a colonic microbial metabolite of adenosine, enhanced the mucosal barrier in a colitis model and exhibited antitumor activity in several cancer models [86,87]. An impairment of cognitive function caused by sleep deprivation was recovered by resveratrol and grape seed polyphenol, but antibiotics disrupted this effect, indicating a role of the colonic microbiota [88]. The following section summarizes information on the production of short-chain fatty acids, amino acid metabolites, and vitamins by the colonic microbiota and their beneficial effects on human health.

#### 2.2.1. Production of Short-Chain Fatty Acids

The colonic microbiota produces enzymes that degrade indigestible carbohydrates such as dietary fiber, inulin, fructo-oligosaccharides, and galacto-oligosaccharides, which are converted to short-chain fatty acids (SCFAs) including propionate, acetate, and butyrate [11]. SCFAs, especially butyrate, are the major energy sources for colonocytes and colonic bacteria. They also regulate host immunity, maintain gut homeostasis, accelerate mineral absorption, enhance gut barrier integrity, and protect the intestinal tract from antimicrobials [89,90,91,92,93,94]. Numerous studies have shown the potential of butyrate for cancer treatment [95,96,97]. For example, mice with intestinal carcinoma showed significantly decreased numbers and sizes of tumors after gavage administration of butyrate-producing bacteria [96]. It is noteworthy that there are contradicting studies [98]. Okumura et al. showed that the butyrate produced by *Porphyromonas asaccharolytica* and *Porphyromonas gingivalis* might induce colorectal tumorigenesis [99]. This discrepancy is associated with factors such as the tumor environment, mutations, and interaction with other metabolites, although the definite mechanisms remain unknown.

However, the health benefits of SCFAs have been described against many diseases [100]. The infection of mice with human sepsis pathogens significantly reduced their number of butyrate-producing bacteria and inhibited the NF-κB and interferon regulatory factor 3 (IRF3) signaling pathways. Levels of NF-κB and IRF3 were recovered by the introduction of butyrate through the transplantation of feces from healthy littermates [101]. Moreover, there is a relationship between SCFAs and neurological disorders such as Alzheimer’s disease, Parkinson’s disease, multiple sclerosis, autism spectrum disorder (ASD), and depression [102,103,104,105]. In a study of children with ASD and transparent dysbiosis, the levels of SCFAs and dopamine metabolites were meaningfully lower compared to those of children without ASD. Intervention with probiotics and a fructo-oligosaccharide increased the levels of SCFAs and dopamine metabolites in children with ASD and markedly ameliorated symptoms of the disease [106]. Furthermore, colonic-microbiota-derived propionic acids are used in the endogenous synthesis of odd-chain fatty acids (OCFAs) in humans and rodents [107]. OCFAs, such as pentadecanoic acid (C15:0) and heptadecanoic acid (C17:0), are produced by rumen gut microbiota and consumed by humans in ruminant fat, such as beef and dairy products [108]. The circulating OCFA level is inversely related to the risk of metabolic and cardiovascular disease [109,110,111,112].

#### 2.2.2. Production of Amino Acid Metabolites

The gut microbiota catabolizes dietary proteins, amino acids, and host-derived endogenous compounds, yielding polypeptides, derivatives of amino acids (i.e., amines, indoles, and phenolic compounds), ammonia, H_2_, CO_2_, and H_2_S [113,114,115]. The production of these metabolites has been reported to be influenced by the proteolytic environment and the composition of the gut microbiota [116,117]. Some of these metabolites exert harmful effects. For instance, in the gut of germ-free mice colonized with the colonic microbiota from patients with celiac disease, *Pseudomonas aeruginosa* catabolized gluten to peptides, which crossed the intestinal barrier and triggered immune responses, whereas *Lactobacillus* spp. from healthy controls degraded these peptides and showed reduced immune responses [118]. However, a range of colonic microbial metabolites derived from amino acids show beneficial effects on diseases. Tryptophan is converted into indole, tryptamine, and indole-3-propionic acid by the colonic microbiota [119,120]. Indole and indole-3-propionic acid stimulate glucagon-like peptide-1 secretion and improve glucose metabolism [121,122,123]. Furthermore, indole and indole-producing bacteria induce neurogenesis in the hippocampus and promote the apoptosis of colorectal cancer cells [55]. Tryptamine improves the gut transit time by increasing colon secretion and attenuates inflammatory bowel disease and autoimmune encephalomyelitis in animal models [124,125,126]. Some colonic microbiota, for example, *Clostridium sporogenes*, are involved in the biosynthesis of serotonin from tryptophan [127,128]. Serotonin is secreted into the GI tract and modulates peristalsis, inflammation, and gut epithelial development.

#### 2.2.3. Production of Vitamins

Vitamins are precursors and/or coenzymes in diverse biochemical pathways, but some essential vitamins cannot be synthesized by humans. The colonic microbiota synthesizes vitamin K_2_ (menaquinone) from K_1_ (phylloquinone) and its derivative K_3_ (menadione), as well as B vitamins such as thiamine, riboflavin, niacin, pantothenic acid, pyridoxine, biotin, folates, and cobalamin [129,130]. The composition of the colonic microbiota can be affected by vitamins, subsequently affecting host immunity and gut homeostasis [131,132]. The relationship between the colonic microbial metabolism of vitamins and host disease is unclear, but a causal connection is proposed [28,133,134,135,136].

Next, we discuss the interactions of phytochemicals with the colonic microbiota and their effects on physiology and disease.

## 3. Therapeutic Effects of Phytochemical Metabolites Produced by the Colonic Microbiota

### 3.1. Phytochemical Metabolites Produced by the Colonic Microbiota

Phytochemicals are bioactive compounds abundant in plant-derived foods such as fruits, vegetables, grains, teas, and mushrooms. The most widely distributed phytochemicals are polyphenols, which have aromatic rings and hydroxyl groups [137]. Based on their structure, polyphenols are classified into flavonoids and non-flavonoids [138]. Flavonoids comprise flavones, flavonols, flavanols, flavanones, isoflavones, and anthocyanidin; non-flavonoids include phenolic acids, stilbenes, and lignans. Phenolic acids are subdivided into hydroxycinnamic acids and hydroxybenzoic acids [139]. Polyphenols have antioxidant, antimicrobial, anti-inflammatory, anticancer, cardioprotective, and neuroprotective activities, making them promising candidates for drug development [19,140,141,142,143,144].

**Figure 3:**
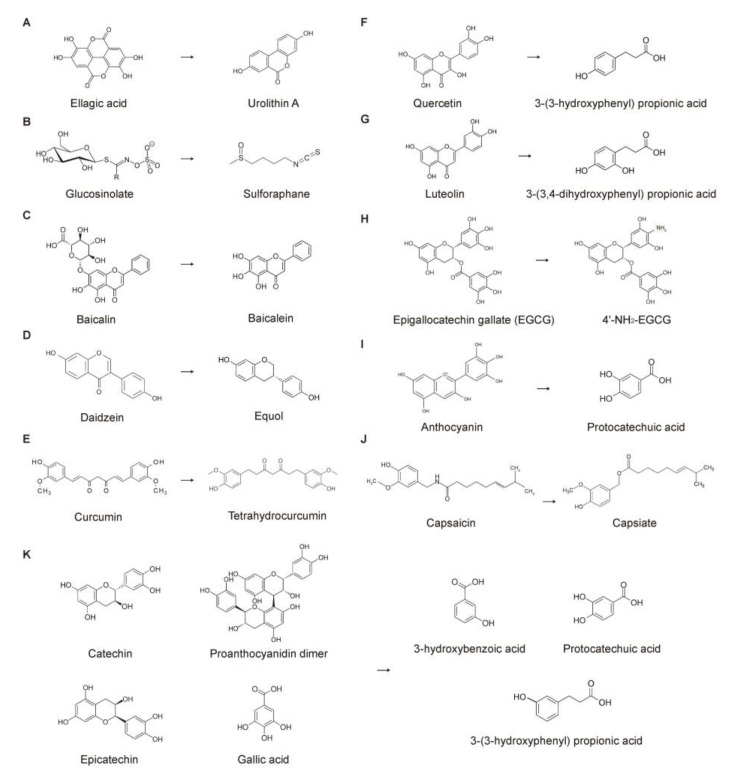
Conversion of phytochemicals by the colonic microbiota. Biotransformation by (**A**) *Clostridiales*, *Ruminococcaceae*; (**B**) *Enterococcus casseliflavus* CP1, *Bacteroides thetaiotaomicron*; (**C**) *Escherichia coli*; (**D**) *Streptococcus intermedius*, Bacteroides ovatus, Ruminococcus productus; (**E**) *Escherichia fergusonii*, *Escherichia coli*; (**F**) *Clostridium, Eubacterium*; (**G**) *Clostridium, Eubacterium*; (**H**) not determined; (**I**) *Bifidobacterium*, *Lactobacillus*; (**J**) not determined; and (**K**) *Bifidobacterium*, *Lactobacillus*, *Clostridium*.

The colonic microbiota degrades undigested polyphenols, enhancing their absorbability [139]. Colonic microbial enzymes mediate polyphenol deglycosylation, demethylation, dehydroxylation, ester cleavage, isomerization, ring fission, and decarboxylation [145,146]. Many phytochemicals are metabolized by different sets of colonic microbiota [147,148,149]. Ellagic acids are metabolized to urolithins by *Clostridium* spp., *Ruminococcaceae, Eubacterium* spp., *Gordonibacter* spp. and *Ellagibacter isourolithinifaciens* [12,150,151]. Daidzeins, isoflavones from soybeans, are converted into equols by *Streptococcus intermedius, Bacteroides ovatus*, and *Ruminococcus productus* [152]. Lignans are modified by various bacteria, e.g., *Clostridium scindens, Eggerthella lenta, Clostridiales*, and *Lactonifactor longoviformis* [153]. This indicates that phytochemical metabolites can be different among individuals eating the same diet because of individual colonic microbiota compositions. Various microbial metabolites derived from phenolic acids and interindividual differences have been reported [154,155]. Figure 3 illustrates several phytochemicals and their colonic microbial metabolites.

### 3.2. Therapeutic Effects of Phytochemical Metabolites

After fermentation by the colonic microbiota, polyphenol metabolites are absorbed by gut epithelial cells and exert beneficial effects on the host. In this section, based on reliable scientific evidence, we provide brief information on some selected phytochemical metabolites produced by the colonic microbiota and their beneficial effects on human diseases.

#### 3.2.1. Urolithins (Metabolites of Ellagitannins and Ellagic Acids)

Ellagitannins—hexahydroxydiphenoic acid esters abundant in berries, walnuts, and pecans—are hydrolyzed to ellagic acids in the gut [156]. Ellagitannins and ellagic acids are metabolized through decarboxylation and dehydroxylation by the colonic microbiota, producing various intermediates (iso-urolithin A abd urolithin C·D·M5 and M6) and urolithin A and B, of which urolithin A is the most bioactive [157,158]. The bacterial taxa that produce urolithin A are unknown, but *Clostridiales* and *Ruminococcaceae* are candidates [157,159,160]. Urolithin A has health-promoting effects on inflammation, neurological disorders, cardiovascular diseases, and cancer [158,161,162,163]. Urolithin A inhibited CD4^+^ T cell proliferation by upregulating microRNA-10a-5p [164]. Urolithin A also suppressed the PI3-K/Akt/NF-κB pathway and proinflammatory factor production in vitro [165]. In Jurkat and K562 leukemia cells, urolithin A and B markedly increased the levels of metabolites that had been shown to promote cell death and induce metabolites associated with oxidative DNA damage [166]. Urolithin A increased senescence activity in a tumor-suppressor protein-p53-dependent manner in human colorectal cancer cells [167]. Furthermore, urolithin A exerted a cardioprotective effect by inhibiting myocardial fibrosis in vitro and in vivo in rats via the Nrf2 pathway [168]. The upregulation of Nrf2 by urolithin A exerted a protective effect in a rat colitis model by increasing tight junction proteins and enhancing gut barrier function [169]. Despite recent evidence showing beneficial effects of urolithin A, pharmacokinetics of urolithin A remains ambiguous. It is known that after absorption, urolithins undergo phase-II metabolism in epithelial cells [170,171]. As a result, conjugated urolithins (mainly glucuronides) are abundantly found in plasma, tissues, and urine. According to recent investigations, urolithin glucuronides had less or a lack of anti-inflammatory capacities compared to urolithins [172,173]. Further research work on the systemic action of urolithins and their conjugated metabolites after entering the circulation is required to clarify the logic of conflicting studies.

#### 3.2.2. Sulforaphane (Metabolite of Glucosinolate)

Glucosinolates are abundant in cruciferous vegetables such as broccoli, cabbage, and kale. Sulforaphane (SFN) is converted from glucosinolate by myrosinase from plants or colonic microbiota such as *Enterococcus casseliflavus* CP1 and *Bacteroides thetaiotaomicron* [174,175]. After absorption, SFN is conjugated with glutathione to form dithiocarbamates through the mercapturic acid pathway [176]. Several studies have shown the presence of dithiocarbamates in plasma, tissues, and urine following SFN intake [177,178]. SFN has anticancer activity via an epigenetic mechanism [179,180]. Epigenetics refers to heritable alterations in gene expression without changes in DNA sequences. These alterations are reversible but can be preserved through mitotic or meiotic division and inherited [181]. Epigenetic modifications make changes in numerous molecular pathways linked to disease [182,183,184]. The major epigenetic mechanisms are DNA methylation, histone modification, and regulation by noncoding RNAs. In SFN-treated HepG2 cells, DNA hypermethylation was observed in transcription factors such as E2F3, THAP1, and ANKHD1, which are involved in cell proliferation, cell cycle progression, and apoptosis [185]. In contrast, the treatment of breast cancer cells with SFN resulted in global DNA hypomethylation, DNA methyltransferase 1 (DNMT1) and DNMT3B deactivation, cell cycle arrest, and senescence [186]. SFN also inhibits histone deacetylases (HDAC) [187,188,189]. SFN stimulated the tumor suppressors miR-9-3 in lung cancer cells by downregulating DNMT3a, HDAC1, HDAC3, HDAC6 [190], and p21 in breast cancer cells [191]. SFN reduced the expression and activity of HDACs and the viability of malignant melanoma cells [192]. The downregulation of HDAC5 by SFN blocked tumor growth in breast cancer cells [193]. In pancreatic cells, SFN decreased the expression of miR30a-3p, which restored connexin 43 expression, gap junction activity, and chemotherapy sensitivity [194]. SFN induced T-cell activation by affecting miR-155-5p and miR-194-5p signaling in the presence of pancreatic-cancer-derived antigens [195]. Furthermore, the tumor-suppressor gene RASAL2 was activated by the SFN-mediated upregulation of miR135b-5p in pancreatic ductal adenocarcinoma cells [196]. The effects of sulforaphane in various diseases are reviewed elsewhere [197,198,199].

#### 3.2.3. Baicalein (Metabolite of Baicalin)

The flavone glycoside baicalin is found in herbs and teas and is hydrolyzed by the colonic microbiota to baicalein [200]. Evidence in the research is scarce, but *E. coli* converts baicalin to baicalein by hydrolysis [201]. The bioactivity and bioavailability of baicalein are greater than those of baicalin [202]. Baicalin and baicalein significantly inhibited the proliferation of HCT-116, HT-29, and SW-480 colon cancer cells, with baicalein having the greater effect; the parent form had a weaker antiproliferative effect on HCT-116 cells and negligible effects in the other two cell types [203]. In *Apc^Min/^*^+^ mice, the number of intestinal tumors and the levels of inflammatory cytokines were decreased by oral baicalein treatment [204]. Moreover, baicalein repressed tumor cell proliferation in a cervical cancer model and induced apoptosis in breast and thyroid cancer models by inhibiting AKT/mTOR signaling [205,206,207]. Baicalein inhibited the activities of HDAC-1 and HDAC-8 and induced proteasomal degradation of HDAC-1, thereby suppressing tumor cell growth and differentiation in in vitro and in vivo models of acute myeloid leukemia [208]. The epigenetic-modification-mediated anticancer activity of baicalein was also observed in a model of type-2-diabetes-induced liver cancer [209]. Baicalein also exerted therapeutic effects in models of Parkinson’s disease, Alzheimer’s disease [202,210,211,212], and metabolic diseases [213,214,215]. The inhibition of the NLRP3 inflammasome by baicalein attenuated osteoarthritis, Parkinson’s disease, acute liver injury, and hyperlipidemia [210,216,217,218,219]. A recent study showed that baicalein exerted substantial antiviral and anti-inflammatory effects in a COVID-19 model both in vitro and in vivo [220].

It is noteworthy that baicalin is poorly absorbed in the small and large intestines, while baicalein is absorbed well and further metabolized to oroxylin A or baicalin again [200,221]. This indicates the critical role of the colonic microbiota in the bioavailability of baicalin and the necessity of further investigations to elucidate the therapeutic effects of baicalein in relation to the interaction between baicalin, baicalein, and the colonic microbiota.

#### 3.2.4. Equol (Metabolite of Daidzein)

Equols are generated from daidzeins, which are abundant in soybean, by the activities of colonic microbiota including *Streptococcus intermedius, Bacteroides ovatus*, and *Ruminococcus productus* [23]. After rapid absorption, equols are found mainly in their conjugated form (i.e., glucuronides) in circulation [222]. The pharmacokinetics of equols are still unclear. However, the bioavailability of equols changes in the enantiomeric forms of equols (i.e., R-equol, S-equol) [223,224]. The role of specific colonic microbiota in producing enantiomeric equols and the mode of action of each equol should be investigated further. As the most bioactive metabolite of daidzein, equol has antioxidant, antitumor, anti-inflammatory, and estrogen-like activities [225,226,227]. In some clinical trials, daidzein efficacy was affected by whether the participants produced equol, although the results were inconsistent [228,229]. The ability to produce equol is dependent on the composition of the colonic microbiota [230,231]. As a phytoestrogen, equol inhibited the tumor growth of B16 cells in a PAP-associated domain containing 5 (PAPD5)-dependent manner. Equol did not affect the tumor growth of PAPD5-ablated B16 cells. Interestingly, equol increased the growth of PAPD5-ablated breast cancer MCF-7 cells with high estrogen receptor expression [232]. Equol exerted a neuroprotective effect via an anti-inflammatory mechanism in vitro and in an animal model [233,234].

#### 3.2.5. Tetrahydrocurcumin (Metabolite of Curcumin)

Tetrahydrocurcumin is a microbial metabolite of the turmeric polyphenol curcumin with equal or higher bioactivity compared to its parent compound. However, the mechanistic pathways underlying the therapeutic effects of tetrahydrocurcumin are unknown [235,236]. *Escherichia fergusonii* and *E. coli* produce tetrahydrocurcumin [237]. Tetrahydrocurcumin is known to exert therapeutic effects in cardiovascular diseases through its antioxidant property [238]. Tetrahydrocurcumin reportedly attenuates diabetes-induced cardiomyopathy by upregulating SIRT1, a histone deacetylase which increases the production of superoxide dismutase [239]. In cerebral ischemic/reperfusion (I/R), the DNA methylation level of CpG regions in the TIMP-2 promoter increases significantly. Tetrahydrocurcumin, a DNMT inhibitor suppressed I/R-mediated DNMT expression [240]. In a mouse model of myocardial I/R, tetrahydrocurcumin prevented heart failure and decreased autophagy and apoptosis by inducing the PI3K/AKT/mTOR pathway in H9c2 cells [241]. The anti-inflammatory activity of tetrahydrocurcumin protected brain microglial cells from lipopolysaccharide-induced injury [242]. Diverse aspects of tetrahydrocurcumin as a potential therapeutic agent are described well in the recent literature [243].

#### 3.2.6. Propionic Acids (Metabolites of Quercetin, Catechin, and Luteolin)

Quercetin, a flavonoid with a bioavailability of <5%, is converted to 3-(3-hydroxyphenyl)propionic acid (3-HPPA) by colonic bacteria including *Clostridium* and *Eubacterium* [244]. It is thought that 3-HPPA is absorbed into the circulation by monocarboxylic acid transporters [245], and 3-HPPA exerts a greater vasodilatory effect than its parent compound [246,247]. Additionally, 3-HPPA reportedly improves bone health [248], and 3-HPPA regulated the cytoplasmic cAMP level, thus inhibiting osteoclastogenesis and osteoclastic bone resorption [249]. Furthermore, 3-HPPA and other propionic acids produced by the colonic microbiota demonstrate neuroprotective activity. α-synuclein is implicated in Parkinson’s disease and multiple system atrophy. It accumulates and spreads in the brain, leading to a worsening of disease symptoms; 3-HPPA prevented aggregation of α-synuclein and related symptoms in vitro [250]. In addition to quercetin, flavan-3-ols such as catechins in red wine are converted to 3-HPPA and other propionic acids by hydrolysis or oxidation by the colonic microbiota [251]. The metabolism of these derivatives in the liver yields diverse metabolites, which cross the blood–brain barrier and exert neuroprotective effects [252,253,254]. In a mouse model and human peripheral blood mononuclear cells, 3(3,4-dihydroxyphenyl) propionic acid from the mixture of Concord grape juice, grape seed extract and transresveratrol, reduced IL-6 expression by regulating DNA methylation in the IL-6 promoter region. IL-6 crosses the blood–brain barrier and influences synaptic plasticity, possibly leading to depressive disorder [255,256]. Among several phytochemicals, luteolin, a phytochemical abundant in citrus and lettuce, is known to be converted to 3(3,4-dihydroxyphenyl) propionic acid by colonic bacteria such as *Clostridium* and *Eubacterium* [145]. 

#### 3.2.7. Other Phytochemical Metabolites Produced by the Colonic Microbiota

Zhang et al. reported that the colonic microbiota enhanced the metabolism of the green tea polyphenol epigallocatechin-3-gallate (EGCG) to 4′-NH_2_-EGCG, which scavenged the neurotoxic reactive carbonyl species methylglyoxal and inhibited the growth of HCT-116 and HT-29 human colon cancer cells [257]. Anthocyanins are a group of flavonoids abundant in blueberries and red wine with beneficial effects but low bioavailability [258]. *Bifidobacterium* and *Lactobacillus* degrade anthocyanins, yielding protocatechuic acid (PCA) and gallic acid [259], which have antioxidant, anticancer, and cardiovascular-protective effects [260,261]. The pharmacokinetics of PCA are still unknown. Nevertheless, Zheng et al. found that PCA is found in circulation in its free form [262]. The regulation of the PI3K/AKT signaling pathway by PCA attenuated type 2 diabetes in a rat model and prevented the apoptosis of human platelets in vitro [263,264]. Furthermore, PCA improved parameters related to the harmful state induced by chronic stress, including serum corticosterone, brain-derived neurotrophic factor, inflammatory cytokines, and TNF-α in a mouse model [265]. Capsiate, an analog of capsaicin from chili pepper, is produced by the colonic microbiota and ameliorates intestinal I/R injury by upregulating glutathione peroxidase 4 and inhibiting ferroptosis [266]. Ho et al. reported the neuroprotective activity of a polyphenol-rich cocoa preparation in gnotobiotic mice colonized by the colonic microbiota from a healthy human donor [267]. Polyphenols in the cocoa preparation included catechin, epicatechin, proanthocyanidin dimers, and gallic acid, which are fermented by *Bifidobacterium, Lactobacillus*, and *Clostridium* [268]. These polyphenols were converted into (+)-catechin, (−)-epicatechin, vanillic acid, 3-hydroxybenzoic acid (3-HBA), 3,4-dihydroxybenzoic acid (3,4-diHBA), and 3-(3-hydroxyphenyl)propionic acid (3-HPPA). Among them, 3-HBA, 3,4-diHBA, and 3-HPPA inhibited the assembly of α-synuclein in vitro and in a *Drosophila* model of Parkinson’s disease. As we have seen above, phytochemical metabolites can modulate various human diseases (Table 1), although the mechanisms need to be clarified further.

Although absorption is an important route by which phytochemicals enter the host, there are other ways. In the next section, we discuss phytochemicals’ modulation of the colonic microbiota and its impacts on various human diseases.

## 4. Modification of the Colonic Microbiota by Phytochemicals and Its Role in Diseases

**Figure 4:**
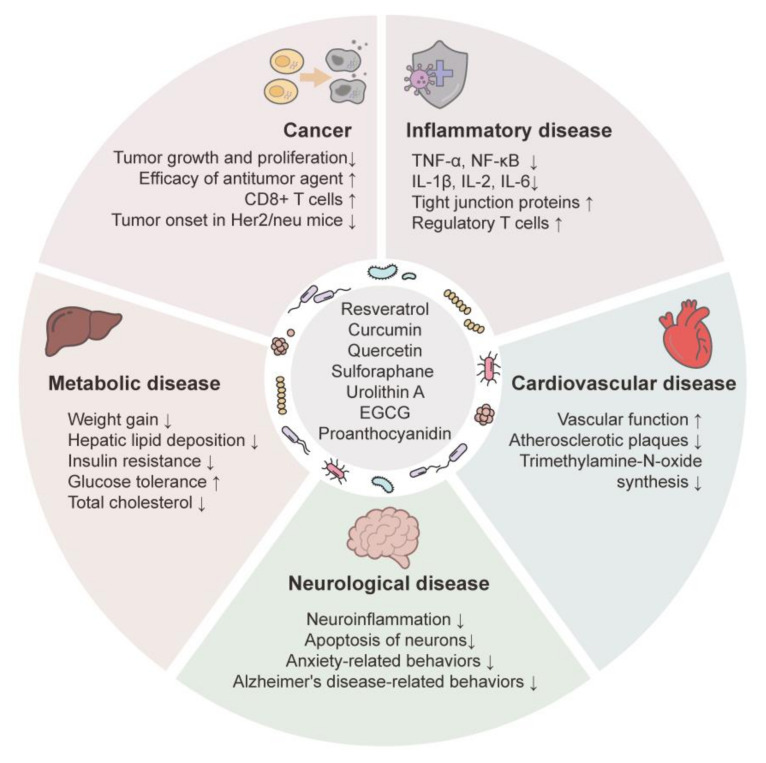
Effects of phytochemicals on diseases via colonic microbiota modulation. Effects of phytochemicals on physiology and pathology indicate associations with the composition and diversity of colonic microbiota. Arrows indicate substantial increases (↑) and decreases (↓).

The colonic microbiota is implicated in a variety of diseases [271,272,273,274,275]. Although little is known of the mechanisms underlying the relationships of polyphenols with the colonic microbiota, recent studies showed that the curative effects of phytochemicals on human diseases were mediated by an alteration in the composition of the colonic microbiota, altering the levels of microbial metabolites and/or the interaction between colonic microbiota and host epithelial cells, ultimately impacting host physiology and pathology. Figure 4 shows the therapeutic effects of phytochemicals in diseases linked to the modification of colonic microbiota.

### 4.1. Modification of the Colonic Microbiota by Phytochemicals in Cancer

According to the WHO and NIH, cancer caused 10 million deaths worldwide in 2020 and imposed an economic burden of more than USD 21 billion on U.S. patients in 2019. Curcumin has antioxidant, anti-inflammatory, antibacterial, and antitumor effects, but its involvement in colonic-microbiota-mediated antitumor activity is unclear [276,277,278,279,280,281]. McFadden et al. suggested a causal link between normalization of the colonic microbiota composition, including an increased abundance of *Lactobacillus*, and the anticancer effects of curcumin in an IL10^−/−^ mouse model of colorectal cancer [281]. In BALB/c mice with hepatocellular carcinoma, Wu et al. used a polyvinylpyrrolidone-based solid dispersion of Zn(II)–curcumin to overcome the low bioavailability of curcumin. The dispersion inhibited tumor growth and protected gut barrier function in a colonic-microbiota-dependent manner, decreasing the abundance of *Firmicutes* and increasing the abundance of *Bacteroidetes* [282]. The Firmicutes-to-Bacteroidetes ratio is a well-known marker for gut dysbiosis. Furthermore, curcumin enhanced the efficacy of 5-fluorouracil, but the effect was absent in antibiotic-treated mice, which also showed a significantly lower absorption of curcumin compared to untreated mice [283]. Green tea polyphenol significantly delayed the onset of estrogen-receptor-negative mammary tumors in Her2/neu transgenic mice, accompanied by a substantial increment in the level of SCFAs and the growth of *Adlercreutzia* and *Lactobacillus* [284]. Green tea catechins induce the proliferation of lactate-producing bacteria [285,286,287], which reportedly protect the gut mucosa [288,289]. Lactate produced by *Bifidobacterium* and *Lactobacillus* spp. Promoted the differentiation and proliferation of intestinal stem cells, thereby protecting the small intestine against radiation-induced injuries [290]. Ming et al. showed that EGCG increased *Turicibacter* and *Lactobacillus* and thereby protected the intestinal tract from radiation-induced injury in a mouse model [291]. Castalagin, a polyphenol from the camu camu berry, shifted the colonic microbial composition and promoted the growth of *Ruminococcaceae*, which was correlated with increased CD8^+^ T cells in the tumor microenvironment [292]. He et al. suggested that sulforaphane improved gut barrier function and anti-inflammatory activity by restoring cancer-related dysbiosis and thereby ameliorated bladder cancer in a mouse model, restoring *Bacteroides fragilis* and *Clostridium cluster I* [269]. Kaempferol is a flavonol found in vegetables and fruits. When fed to *Apc^Min/^*^+^ mice, kaempferol reduced intestinal polyps and proinflammatory cytokines and increased the levels of enzymes involved in bile acid synthesis (i.e., CYP8B1 and CYP27A1) [293]. Kaempferol reduced *Anaerostipes, Desulfovibrio* and *Helicobacter*, which are abundant in the colon of colorectal cancer patients, and downregulated the secondary bile acid synthesis pathways. The latter were correlated with decreased levels of bile-acid-producing bacteria such as *Clostridium lavalense, Eubacterium desmolans*, and *Eubacterium rectale.*

### 4.2. Modification of the Colonic Microbiota by Phytochemicals in Metabolic Diseases

Colonic-microbiota-mediated therapeutic effects of phytochemicals are especially noticeable in metabolic diseases. Rinott et al. compared the effect of diet on the colonic microbiota and the influence thereof on weight loss and glycemic control [294]. Autologous fecal microbiota transplantation showed that a polyphenol-rich Mediterranean diet induced the proliferation of beneficial bacteria such as *Bacteroides massiliensis* and *Paraprevotella clara,* attenuating weight gain and sustaining glycemic control. Oat is rich in β-glucans, which promote metabolic function, particularly cholesterol homeostasis and glucose control [295,296]. In a clinical trial involving hypercholesterolemic patients, oat consumption reduced the levels of total cholesterol and low-density lipoprotein cholesterol, which was correlated with the abundance of butyrate-producing *Akkermansia muciniphila* and *Roseburia* [297]. Resveratrol exerts beneficial effects on metabolic diseases [298,299,300,301,302]. When administered by gavage to high-fat diet (HFD)-fed mice, resveratrol decreased *Desulfovibrio* and *Lachnospiraceae_NK4A136_group*, which are related to obesity, and fecal transplantation from resveratrol-treated mice to HFD-fed mice increased the expression of tight junction proteins and the mRNA levels of fatty-acid oxidation and thermogenic genes [300]. Curcumin promoted the growth of SCFA-producing species such as *Bacteroides, Akkermansia, Parabacteroides, Alistipes*, and *Alloprevotella* and induced weight loss in mice with HFD-induced obesity and hepatic steatosis. PICRUSt analysis showed that altered bacterial gene expression was related to carbohydrate and lipid metabolism, glycolysis, gluconeogenesis, and bile secretion [303]. In a non-alcoholic fatty liver disease model, the proliferation of SCFA-producing bacteria such as *Butyricicoccus* and *Lactobacillus* was correlated with disease amelioration [304]. Curcumin ameliorated uric acid nephropathy in a rat model. Curcumin decreased *Escherichia-Shigella* and *Bacteroides*, which produce enzymes for the synthesis of indole, p-cresol, tryptophan, and tyrosine [305]. It should also be noted that p-cresol and some of indole derivatives, such as indole-3-acetic acid and indoxyl sulfate, are uremic toxins converted from tryptophan and tyrosine by the colonic microbiota [119,306]. The garlic phytochemical allicin altered the composition of the colonic microbiota by increasing the abundance of *Bifidobacterium* and *Lactobacillus*. The transplantation of an allicin-treated colonic microbiota to HFD-fed mice decreased adiposity, maintained glucose homeostasis, and ameliorated hepatic steatosis [307]. Similar anti-obesity effects and the modulation of the colonic microbiota were observed in glutamate-induced obese mice fed quercetin [308]. In combination with inulin (a non-digestible polysaccharide), isoquercetin, a glucoside of quercetin, maintained homeostasis of glucose metabolism and insulin resistance by normalizing the composition of the colonic microbiota, which is disrupted by a high-fat diet [309]. Proanthocyanidin from wild blueberry attenuated HFD-induced obesity by enriching *Akkermansia muciniphila*, leading to the proliferation of goblet cells [310]. Proanthocyanidin from grape seed extract increased the abundance of *Prevotella, Clostridium XIVa*, and *Roseburia*, which are butyrate-producing bacteria, leading to the amelioration of macrophage infiltration and decreased inflammatory cytokine levels [310,311].

### 4.3. Modification of the Colonic Microbiota by Phytochemicals in Inflammatory Diseases

In a mouse model of intestinal inflammation, EGCG modulated inflammation mediated by the colonic microbiota [312]. The oral administration of EGCG increased the abundance of the beneficial bacteria *Akkermansia* and was significantly correlated with SCFA production and IL-8 downregulation in colitis mice. This anti-inflammatory effect was supported by fecal microbiota transplantation from EGCG-treated mice to colitis mice [313]. In mice with HFD-induced obesity, EGCG not only increased the abundance of *Akkermansia* and other beneficial colonic bacteria, including *Bacteroides* and *Parasutterella*, but also regulated the levels of inflammatory factors such as IgA, major histocompatibility complex-II, and signaling molecules related to the NF-κB pathway, thus maintaining homeostasis [314]. In an animal model of lipopolysaccharide-induced systemic inflammation, EGCG suppressed the levels of inflammatory factors, including TNF-α and several interleukins, and reversed dysbiosis by decreasing the abundance of *Enterobacteriales* [315].

Resveratrol increased the abundance of *Lactobacillus* spp., especially *Lactobacillus reuteri*, and the survival rate by inhibiting proinflammatory cytokines and stimulating anti-inflammatory cytokines in mice with lethal acute respiratory distress syndrome caused by staphylococcal enterotoxin B [302]. Furthermore, resveratrol aids dysbiosis recovery by increasing *Ruminococcus gnavus* and *Akkermansia muciniphila*. The anti-inflammatory effect was supported by fecal transplantation from resveratrol-treated mice to colitis mice [316]. In colitis mice, Li et al. showed that the abundance of *Lactobacillus* and *Bifidobacterium* was recovered by resveratrol and was negatively correlated with the levels of IL-1β, IL-2, IL-6, TNF-α, and granulocyte-macrophage colony-stimulating factor [317]. In mice with allergic asthma, resveratrol increased the abundance of *Bacteroides* in the gut [318]. Butyrate produced by the colonic microbiota induced anti-inflammatory regulatory T cells, thus ameliorating asthma.

Espín et al. suggested that the anti-inflammatory activity of urolithin A was facilitated by *Lactobacillus* and *Bifidobacterium* in a rat model of colitis [319]. Hu et al. reported that PCA increased the abundance of *Roseburia* and *Desulfovibrio* and reduced the serum levels of IL-1β, IL-2, and IL-6 in lipopolysaccharide-treated piglets [320]. Vanillic acid, a metabolite of the anthocyanin cyanidin 3-glucoside, exerted an anti-inflammatory effect by reducing *Prevotella 7*, which showed a positive correlation with IL-1b, IL-2, and IL-6 [321].

Curcumin attenuated inflammation in BALB/c mice with colitis induced by dextran sulfate sodium [322]. In this study, a nano form of curcumin suppressed NF-κB activation, mRNA levels of inflammatory mediators, and the number of CD4^+^ Foxp3^+^ regulatory T-cells. These effects were accompanied by an increased abundance of *Clostridium* cluster IV and XIVa, which reportedly produce butyric acid and are implicated in the induction of mucosal regulatory T cells [323].

### 4.4. Modification of the Colonic Microbiota by Phytochemicals in Cardiovascular Diseases

The alteration of the colonic microbiota by phytochemicals is linked to cardiovascular diseases. The aronia berry is rich in polyphenols such as anthocyanins and exerts health-promoting effects [324]. In a clinical trial with healthy adults, aronia berry extract significantly improved endothelial function, which was correlated with an increased abundance of butyrate- and propionic-acid-producing colonic microbiota such as *Dialister, Phascolarctobacterium*, and *Roseburia* [325]. Propionic acid is known to exert a cardioprotective effect [326,327]. In a mouse model, resveratrol attenuated atherosclerosis by increasing *Prevotella, Akkermansia*, *Lactobacillus*, and *Bifidobacterium*, indicating a correlation with the inhibited microbial synthesis of trimethylamine-N-oxide (TMAO), a risk factor for atherosclerosis produced by the colonic microbiota from dietary choline [328]. Geraniin, an ellagitannin in *Geranium thunbergia* and other plants, enriched *Bacteroides* in C57BL/6J ApoE^(−/−)^ mice and suppressed the plasma level of TMAO, resulting in decreased levels of proinflammatory cytokines and lipid uptake in macrophages [329]. In ApoE^(−/−)^ mice, peanut skin extract, containing procyanidins, catechin, epicatechin and EGCG, reduced atherosclerotic plaques and the levels of TNF-α and IL-6, effects correlated with the abundance of beneficial bacteria such as *Roseburia*, *Akkermansia*, and *Bifidobacterium* [330].

### 4.5. Modification of the Colonic Microbiota by Phytochemicals in Neurological Diseases

There is a relationship between the colonic microbiota and neurological diseases such as Alzheimer’s disease, Parkinson’s disease, depression, anxiety, attention deficit hyperactivity disorder (ADHD), autism spectrum disorder, and schizophrenia [331,332,333,334,335,336,337]. Given the effects of phytochemicals on the colonic microbiota, they are likely to influence neurological disorders via the gut–brain axis. [38]. In a mouse model of dextran-sulfate-sodium-salt-induced anxiety, curcumin reversed anxiety-related harmful effects, such as anxiety-related behaviors, and the phosphatidylcholine level in the prefrontal cortex by upregulating *Muribaculaceae* [338]. In a mouse model of Alzheimer’s disease and SH-SY5Y cells, quercetin significantly attenuated AD-related parameters and symptoms. This attenuation was correlated with the abundance of *Barnesiella, Lactobacillus*, and *Parasutterella* [339]. Table 2 summarizes information on the modulation of colonic microbiota by phytochemicals and their therapeutic effects on human diseases.

The influence of other factors (i.e., diet, antibiotics, acute stress, and exercise) must also be validated. Large clinical trials are necessitated by the marked inter-individual variability in colonic microbiota composition and activity in vitro and in vivo.

## 5. Conclusions

The gut microbiota constantly affects host health by digesting the host’s diet and producing various beneficial metabolites including SCFAs, indole, tryptamine, serotonin, and even essential vitamins such as Bs and K2. The gut microbiota also works as an essential degrader and enhances the bioactivity and bioavailability of many phytochemicals such as ellagitannin, curcumin, baicalin, and quercetin [158,202,247,342]. Following degradation by the gut microbiota, phytochemical metabolites have an impact on cellular signaling pathways (i.e., Nrf2 pathway, NF-κB pathway, PI3K/AKT/mTOR pathway, and the secondary bile acid synthesis pathway, etc.) [165,168,205,293]. These metabolites have shown therapeutic activities in wide range of diseases such as cancer, inflammatory diseases, cardiovascular diseases, Alzheimer’s disease, Parkinson’s disease, and depression.

Phytochemicals also affect the composition and diversity of the colon microbiota, thereby playing very important roles in the amelioration of various diseases. Phytochemicals themselves also affect the host by influencing the composition and diversity of the colon microbiota [343,344,345,346,347,348]. In other words, phytochemicals have been reported to enrich the beneficial microflora (i.e., butyrate-producing bacteria) that produce metabolites, playing a role as disease-ameliorating signaling molecules or enzymes [139]. However, the mechanism remains unknown in many cases; therefore, the profiling and functional analysis of the colonic microbiome and microbial metabolites to validate the underlying mechanism is a complex but high priority. It should not be overlooked that individual differences in the composition of the colonic microbiota affect the host’s phytopharmacology [20,349]. The influence of other factors (i.e., diet, antibiotics, acute stress, and exercise) must also be validated. Significant interindividual variability in the composition of the colonic microbiome and activity in vitro and in vivo necessitates large-scale clinical trials.

Collectively, this review presents up-to-date information on the role of the colon microbiome in human health which could be indispensable for future preclinical and clinical investigations.

## Figures and Tables

**Table 1:** Phytochemical metabolites and their effects on human disease.

Phytochemical	Metabolites	Diseases	Effects of Metabolites	Study Design	References
Ellagitannin	Urolithin A	Leukemia	Promoted apoptosis and inhibited proliferation of leukemic cells	in vitro	[166]
		Colorectal cancer	Increased senescence of cancer cells	in vitro	[167]
		Myocardial infarction	Inhibited proliferation of myocardial fibrosis	in vitro and in vivo	[168]
		Colitis	Reinforced gut barrier function	in vitro and in vivo	[169]
Glucosinolate	Sulforaphane	Liver cancer	Promoted apoptosis and inhibited proliferation of human liver cancer cells	in vitro	[185]
		Breast cancer	Induced senescence and apoptosis of human breast cancer cells; stimulated tumor suppressors; Inhibited tumor growth	in vitro	[186,191,193]
		Lung cancer	Stimulated tumor suppressors	in vitro	[190]
		Malignant melanoma	Reduced melanoma cell viability	in vitro	[192]
		Pancreatic cancer	Enhanced gap junction activity and chemotherapy sensitivity; improved dendritic cell activity; activated tumor suppressor gene	in vitro and in vivo	[194,195,196]
		Bladder cancer	Inhibited tumor progression	in vivo	[269]
Baicalin	Baicalein	Colon cancer	Inhibited proliferation of cancer cells	in vitro, in vivo and in silico	[203,204]
		Cervical cancer	Inhibited proliferation of cancer cells	in vitro and in vivo	[205]
		Thyroid cancer	Inhibited proliferation of cancer cells	in vitro	[206]
		Breast cancer	Inhibited proliferation of cancer cells	in vitro and in vivo	[207]
		Myeloid leukemia	Inhibited proliferation of cancer cells	in vitro and in vivo	[208]
		Cutaneous T-cell lymphomas	Inhibited proliferation of cancer cells	in vitro and in vivo	[270]
		Liver cancer	Inhibited tumor growth and progression	in vitro and in vivo	[209]
		Parkinson’s disease	Protected substantia nigra dopamine neuron by preventing inflammation and apoptosis	in vivo	[202,210,211,217]
		Alzheimer’s disease	Inhibited aggregation of hTau40	in vitro	[212]
		Hyperuricemic nephropathy	Exhibited nephroprotective effects	in vivo	[213,214]
		Osteoarthritis	Inhibited disease progression and improved cartilage metabolism	in vivo	[216]
		Acute liver injury	Improved liver function	in vitro and in vivo	[218]
		Pancreatitis	Inhibited pyroptosis and inflammation	in vivo	[219]
Daidzein	Equol	Melanoma	Inhibited tumor growth	in vitro and in vivo	[232]
		Breast cancer	Promoted tumor growth	in vitro	[232]
		Depression	Decreased inflammation and normalized neurotransmitter levels	in vivo	[233]
		Parkinson’s disease	Decreased neurotoxicity	in vitro and in vivo	[234]
Curcumin	Tetrahydrocurcumin	Cardiomyopathy	Decreased oxidative stress and fibrosis	in vivo	[239]
		Ischemic/reperfusion injury	Promoted mitochondria function; Prevented apoptosis	in vitro and in vivo	[240,241]
		Brain inflammation	Reduced inflammation and oxidative stress	in vitro	[242]
Quercetin and Catechin	3-(3-hydroxyphenyl) propi-onic acid		Decreased blood pressure	Human study, in vivo	[246,247]
			Promoted growth and inhibited senescence of osteoblastic cell	in vivo	[248]
			Inhibited osteoclastogenesis	in vitro	[249]
		Parkinson’s disease	Prevented aggregation of α-synuclein	in vitro	[250]
		Alzheimer’s disease	Prevented neurotoxic accumulation of β-amyloid	in vitro and in vivo	[254]
Epigallocatechin-3-gallate	4′-NH2-EGCG	Colon cancer	Eliminated toxic compounds and inhibited growth of cancer cells	in vivo	[257]
Anthocyanin	Protocatechuic acid		Inhibited platelet apoptosis	in vitro	[264]
		Type 2 diabetes	Improved glucose metabolism and insulin sensitivity	in vivo	[263]
		Depressive disorder from chronic stress	Attenuated depressive behaviors and inflammation	in vivo	[265]
Capsaicin	Capsiate	Intestinal ischemic/reperfusion injury	Reduced IRI by inhibiting ferroptosis	in vitro and in vivo	[266]
Cocoa polyphenols	3-hydroxybenzoic acid, 3,4-dihydroxybenzoic acid, 3-(3-hydroxyphenyl) propionic acid	Parkinson’s disease	Inhibited α-synuclein assembly	in vitro and in vivo	[267]

Abbreviations: EGCG—epigallocatechin-3-gallate. IRI—ischemic/reperfusion injury.

**Table 2:** Modulation of colonic microbiota by phytochemicals and their effects on diseases.

Diseases	Phytochemicals or Diets	Alteration of Colonic Microbiota	Therapeutic Effects	Model	Reference
Colorectal cancer	Curcumin	*Lactobacillus* ↑; Recovery of dysbiosis	Survival rate ↑ Hyperplasia ↓ Aberrant localization of β-catenin ↓	Mouse	[281]
	Kaempferol	*Anaerostipes; Desulfovibrio; Helicobacter; Clostridium lavalense; Eubacterium desmolans* *↓*	Intestinal polyps ↓ Proinflammatory cytokines ↓ Bile-acid-synthesizing enzymes ↑	Mouse	[293]
Hepato-cellular carcinoma	Zn(II)-curcumin	Recovery of dysbiosis	Tumor growth ↓ Zinc homeostasis↑ Chemosensitizing ↑	Rat	[282]
	Curcumin	*Bifidobacterium; Lactobacillus* ↑	Tumor proliferation ↓ Chemosensitivity to 5- Fluorouracil ↑	Mouse	[283]
Breast cancer	Green tea polyphenol	*Adlercreutzia; Lactobacillus;* Lachnospiraceae *↑*	Delayed onset of the disease	Mouse	[284]
Radiation-induced intestinal injury	EGCG	*Turicibacter; Lactobacillus* ↑ Recovery of dysbiosis	Crypt cell Proliferation ↑ Intestinal stem cell survival ↑	Mouse	[291]
Fibrosarcoma	Castalagin	*Ruminococcaceae; Alistipes; Christensenellaceae R-7* group; *Paraprevotella* ↑	CD8^+^ T cells ↑ Tumor size ↓	Mouse	[292]
Bladder cancer	Sulforaphane	*Clostridium cluster I*	Submucosal capillary growth ↓ IL-6, SIgA ↓ Normalization of colon tissue and tight junction proteins	Mouse	[269]
Obesity	(polyphenol-rich) Mediterra-nean diet	*Bacteroides massiliensis; Paraprevotella clara* *↑*	Weight gain ↓ Sustained glycemic control	Human	[294]
	Resveratrol	*Desulfovibrio; Lachnospiraceae_NK4A136* *↓*	Tight junction proteins ↑ Transcription of lipid oxidation- and thermogenic- related genes	Mouse	[300]
	Quercetin	*Firmicutes* *↓* *Lachnospiraceae; Ruminicoccaceae* *↑*	Eosinophilic neurons ↓ Weight gain ↓ Hepatic lipid deposition↓ Mucus secretion ↑	Mouse	[308]
	Isoquercetin (with inulin)	*Lachnospiraceae* *↑* *Rikenellaceae* *↓*	Weight gain ↓ Insulin resistance ↓ Improved Glucose tolerance	Mouse	[309]
	Proantho-cyanidin	*Adlercreutzia equolifaciens; Akkermansia muciniphila* *↑*	Number of goblet cells ↑ Improved glucose tolerance	Mouse	[310]
	Proantho-cyanidin	*Clostridium XIVa; Roseburia; Prevotella* *↑*	Pro-inflammatory cytokines ↓ JNK and NF-κB signaling ↓ Insulin resistance ↓	Mouse	[311]
	EGCG	*Akkermansia; Bacteroides; Parasutterella* *↑*	Pro-inflammatory cytokines ↓ NF-κB signaling ↓	Mouse	[314]
Obesity and hepatic steatosis	Curcumin	*Bacteroides; Akkermansia; Parabacteroides; Alistipes; Alloprevotella* *↑*	Weight gain ↓ Insulin resistance ↓ Glucose tolerance ↑ Lipid accumulation ↓ Circulating LPS levels ↓	Mouse	[303]
	Allicin	*Bifidobacterium; Lactobacillus* *↑*	Adiposity ↓ Glucose homeostasis	Mouse	[307]
Non-alcoholic fatty liver disease	Curcumin	*Butyricicoccus; Lactobacillus* *↑*	Low-density lipoprotein cholesterol ↓ Lipid accumulation ↓ Tight junctions ↑	Mouse	[304]
Hyper-cholesterol	Oat	*Akkermansia muciniphila; Roseburia* *↑*	Total cholesterol ↓ Low-density lipoprotein cholesterol ↓	Human	[297]
Uric acid nephropathy	Curcumin	*Escherichia-Shigella; Bacteroides* *↓*	Serum level of uric acid↓ Detrimental histopathologic changes in kidney ↓	Rat	[305]
Intestinal inflammation	EGCG	Recovery of dysbiosis	Pro-inflammatory cytokines ↓ Tight junction proteins ↑ Superoxide dismutase ↑ Glutathione peroxidase ↑	Mouse	[312]
	EGCG	*Enterobacteriales ↓* Recovery of dysbiosis	Pro-inflammatory cytokines ↓	Mouse	[315]
	Protocate-chuic acid	*Roseburia; Desulfovibrio*	Pro-inflammatory cytokines ↓ Tight junction proteins ↑	Piglet	[320]
	Vanillic acid	Prevotellaceae ↓	Pro-inflammatory cytokines ↓ Tight junction proteins ↑	Piglet	[321]
Acute respiratory distress syndrome	Resveratrol	*Lactobacillus reuteri* *↑*	Pro-inflammatory cytokines ↓ Anti-inflammatory cytokines ↑	Mouse	[302]
Colitis	EGCG	*Akkermansia* *↑*	SCFA ↑ IL-8 ↓	Mouse	[313]
	Resveratrol	*Ruminococcus gnavus; Akkermansia* *↑*	Pro-inflammatory cytokines ↓	Mouse	[316]
	Resveratrol	*Lactobacillus; Bifidobacterium* *↑*	Pro-inflammatory cytokines ↓ Granulocyte macrophage colony stimulating factor ↓	Mouse	[317]
	Urolithin A	*Lactobacillus; Bifidobacterium* *↑*	Pro-inflammatory cytokines ↓	Rat	[319]
	Curcumin	*Clostridium cluster IV and XIVa* *↑*	CD4^+^ Foxp3+ regulatory T cells ↑	Mouse	[322]
Allergic asthma	Resveratrol	*Bacteroides; Akkermansia* *↑*	Pro-inflammatory cytokines ↓ Tight junction proteins ↑	Mouse	[318]
Healthy condition	Aronia berry extract	*Anaerostipes; Bifidobacterium* *↑*	Vascular function ↑	Human	[325]
Atherosclerosis	Resveratrol	*Bacteroides; Lactobacillus; Bifidobacterium; Akkermansia* ↑	Trimethylamine-N-oxide synthesis ↓	Mouse	[328,340]
	Geraniin	*Bacteroides; Alloprevotella; Alistipes* *↑*	Trimethylamine-N-oxide synthesis ↓ Lipid uptake in macrophages ↓ Pro-inflammatory cytokines ↓	Mouse	[329]
	Peanut skin extract	*Roseburia; Akkermansia; Bifidobacterium* ↑	Atherosclerotic plaques ↓	Mouse	[330]
Anxiety	Curcumin	*Muribaculaceae* ↑	Anxiety-related behaviors ↓ Phosphatidylcholine in prefrontal cortex ↓	Mouse	[338,341]
Alzheimer’s disease	Quercetin	*Barnesiella; Lactobacillus; Parasutterella ↑* Recovery of dysbiosis	Spatial Memory Impairment ↓ Neuroinflammation ↓ Apoptosis of hippocampus neurons ↓	Mouse	[339]

Abbreviations: EGCG—epigallocatechin-3-gallate; IL—interleukin; JNK—c-Jun-N-terminal kinase; LPS—lipopolysaccharide; SIgA—secretory immunoglobulin A; Arrows indicate substantial increases (↑) and decreases (↓).

## Data Availability

No new data were created or analyzed in this study. Data sharing is not applicable to this article.
